# Comparison of power Doppler and thermography for the selection of thyroid nodules in which fine-needle aspiration biopsy is indicated*

**DOI:** 10.1590/0100-3984.2014.0111

**Published:** 2016

**Authors:** Maria Lucia D'Arbo Alves, Manoel Henrique Cintra Gabarra

**Affiliations:** 1 PhD, Professor of Medicine at the Universidade de Ribeirão Preto (Unaerp), Ribeirão Preto, SP, Brazil.; 2 Doctoral Student in Environmental Technology, Professor of Engineering at the Universidade de Ribeirão Preto (Unaerp), Ribeirão Preto, SP, Brazil.

**Keywords:** Thyroid gland, Thyroid nodule/blood supply, Ultrasonography, Doppler, Thermography

## Abstract

**Objective:**

To compare two methods-power Doppler and thermography-for the analysis of nodule vascularization and subsequent selection of nodules to be biopsied.

**Materials and Methods:**

A total of 510 subjects with thyroid nodules were analyzed by power Doppler and submitted to fine-needle aspiration biopsy (FNAB). Thirty-seven patients were submitted to nodule excision (29 due to carcinoma or suspected carcinoma and 8 by patient choice). Among those patients, power Doppler had raised the suspicion of malignancy in 39 lesions, compared with 48 for FNAB. Another group, comprising 110 patients, underwent thermography, which raised the suspicion of malignancy in 124 thyroid nodules, as did FNAB. Malignant nodules were excised in all 110 of those patients (95 underwent nodulectomy and 15 underwent thyroidectomy), malignancy being confirmed by intraoperative examination of frozen biopsy samples.

**Results:**

In relation to the FNAB findings, the sensitivity, specificity, positive predictive value, negative predictive value, and accuracy of power Doppler were 95.16%, 23.52%, 96.22%, 16.70%, and 89.51%, respectively, compared with 100%, 58.06%, 87.73%, 100%, and 89.51%, respectively, for thermography.

**Conclusion:**

Thermography was more precise than was power Doppler for the selection of thyroid nodules to be biopsied.

## Abstract (pt)

**Objetivo:**

Comparar dois métodos de análise de vascularização de nódulos - power Doppler e termografia - na seleção para submissão a biópsia.

**Materiais e Métodos:**

Foram analisados 510 pacientes portadores de nódulos tireoidianos pelo power Doppler, todos submetidos a punção aspirativa por agulha fina (PAAF). Desses pacientes, 37 tiveram seus nódulos ressecados (29 por carcinoma ou suspeito para carcinoma e 8 por decisão pessoal), totalizando 39 lesões suspeitas para malignidade ao power Doppler e 48 à PAAF. Em outros 110 pacientes foram detectados, pela termografia e PAAF, 124 nódulos tireoidianos, que foram submetidos a excisão (95 casos por nodulectomia e 15 casos por tireoidectomia total) e tiveram malignidade confirmada no exame de congelação intraoperatório.

**Resultados:**

O power Doppler e a termografia apresentaram, respectivamente, sensibilidades de 95,16% e 100%, especificidades de 23,52% e 58,06%, valores preditivos positivos de 96,22% e 87,73%, valores preditivos negativos de 16,70% e 100% e acurácias de 91,83% e 89,51%, quando comparados com a PAAF.

**Conclusão:**

A termografia foi mais precisa que o power Doppler na seleção de nódulos tireoidianos com indicação de biópsia.

## INTRODUCTION

Thyroid nodule (TN) represents an increase in thyroid volume with excessive growth and structural or functional transformation of one or more areas of the thyroid parenchyma that are not associated with the presence of processes related to autoimmune or inflammatory diseases^(1)^. TN can be single or multiple; solid, cystic, or mixed; and functional (hot nodules) or not. The prevalence of TN is 5-7% when determined by palpation, 13-67% when determined by ultrasound, and 30-60% at autopsy^(2)^. The adoption of high resolution ultrasound has revealed an even higher prevalence of TN, and the current suggestion is that all patients with palpable TN should be submitted to ultrasound examination^(3,4)^. The agents that stimulate the onset of TN can be of environmental origin (radiation, smoking, iodine deficiency, medications, stress, infections, or pregnancy) or of constitutional origin (family history, female gender, or age)^(5)^.

The clinical diagnosis of TN is usually based on inspection and palpation of the thyroid, which can be impaired if the nodule is located in a retrosternal position or if the patient is obese. Normally, the thyroid is not visible. The important factor is the exclusion of malignancy. Most TNs are of a benign nature, requiring no surgical intervention. The risk of malignancy and the presence of multiple nodules do not seem to increase during the evolution of TN. Approximately 5-10% of TNs are malignant^(6)^, and the chance of malignancy should be properly excluded by means of specific exams^(7)^.

Because of its superficial location, the thyroid is easily accessible to ultrasound or thermographic exploration and to aspiration biopsy. Imaging studies provide more precise information regarding TN volume, extent, and characteristics.

Fine needle aspiration biopsy (FNAB) is considered the most sensitive preoperative method for the identification of malignant TN, whereas ultrasound is the test most frequently used because of its practicality and its utility in guiding a biopsy of the nodule^(8)^.

A biopsy is indicated for solid or mixed but preponderantly solid nodules larger than 1.0 cm in diameter or for those smaller than 1.0 cm if they show characteristics suggestive of malignancy or if the patient has a history suggesting an increased risk for thyroid carcinoma (neck or whole body irradiation or a family history of thyroid cancer)^(9,10)^. Nodules smaller than 5 mm in diameter should not be punctured even when they show suspicious characteristics on ultrasound examination, due to the high rate of false-positive results^(11)^.

The use of ultrasound for the evaluation of the cervical region has led to the detection of large numbers of nonpalpable nodules and to controversies about whether they should all be analyzed for malignancy^(7)^. Using power Doppler ultrasound to evaluate the characteristics of TN vascularization and its association with malignancy is a possible alternative^(12)^. Power Doppler studies for the diagnosis of malignant thyroid tumors have demonstrated that the risk of malignancy is greater when the vascularization is predominantly or exclusively intranodular or central^(13,14)^. However, Faria et al.^(15)^ observed that a significant proportion of papilliferous carcinomas do not show intranodular vascularization, suggesting that Doppler ultrasound is the best method for the selection of TNs to be biopsied and for guidance during FNAB.

The thyroid can also be studied by thermography, a technique based on the measurement of skin temperature in a determined region. The human skin behaves like a large infrared emitter similar to a black-body radiator and does not emit reflected radiation into the environment. Measurements of the radiation emitted by the skin can be directly converted to temperature values. The local skin temperature is influenced by vascular changes, variations in biological activity, modifications of conduction tissue, and endocrine factors. The growth and biological activity of tissue produce stronger emissions than do inactive tissue, and fluid collections produce less heat. Thermography produces a characteristic and easily recognizable pattern^(16-19)^, as well as determining the ranges of thermal difference that can suggest malignancy of a TN^(18,19)^.

The objective of the present study was to compare power Doppler ultrasound and thermography for the selection of TNs to be biopsied.

## MATERIALS AND METHODS

We analyzed 510 patients using a power Doppler ultrasound imaging system (8000 EX; Samsung Medison Co., Seoul, Korea) with three multifrequency probes. Of those 510 patients, 478 were women and 32 were men. Among the women, ages ranged from 18 to 78 years (mean, 50.2 years; median, 50.5 years), whereas they ranged from 23 to 70 years (mean, 57.2 years; median, 50.0 years) among the men.

A total of 1078 TNs were identified (1021 in women and 57 in men). Of those, 868 were submitted to FNAB (829 for females and 39 for males), 35 women and 4 men being submitted to total or partial thyroidectomy.

According to the classification system devised by Lagalla-Chammas^(13,14)^, the TNs studied by power Doppler were divided into classes 2 and 3 (suggesting benign lesions) and classes 4 and 5 (suggesting lesions suspected of malignancy). These findings were correlated with those obtained by FNAB according to the Bethesda classification^(20)^.

**Figure 1:**
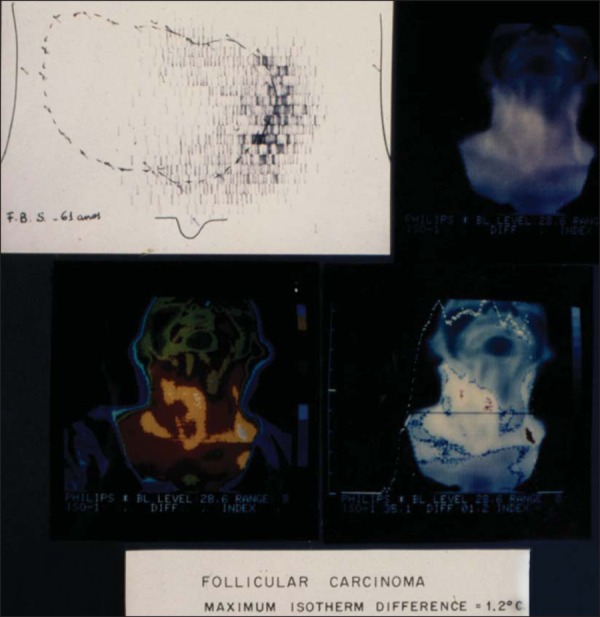
Scintigraphy and thermography of a thyroid nodule (follicular carcinoma).

**Figure 2:**
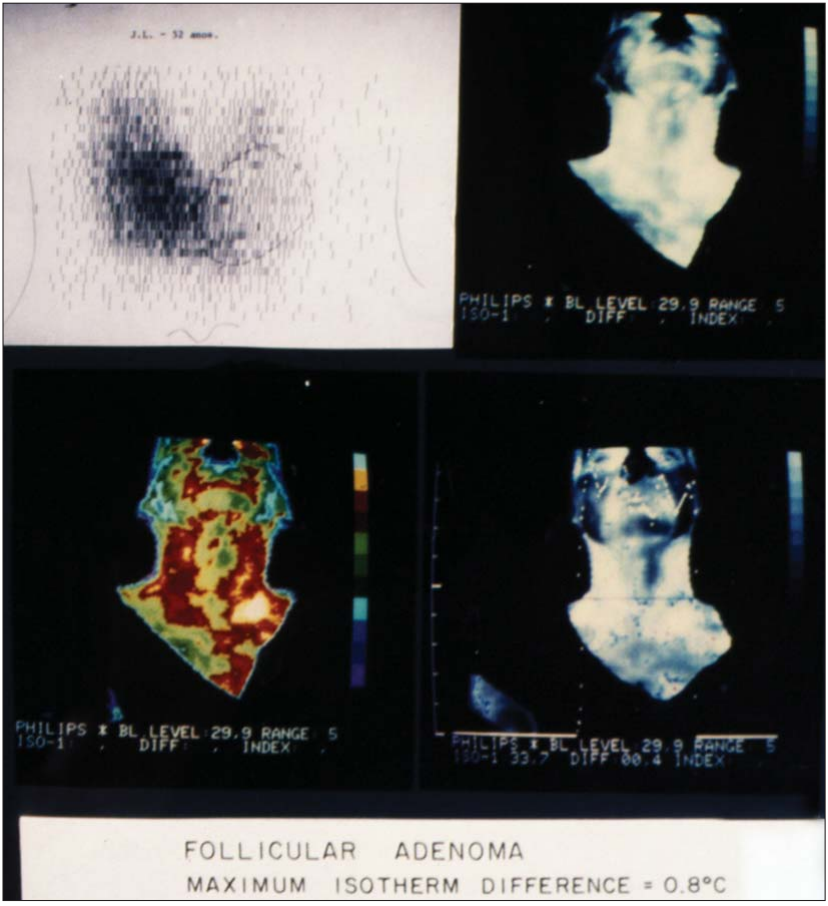
Scintigraphy and thermography of a thyroid nodule (follicular adenoma).

The thermographic study was performed with a digital (dynamic) thermography imaging system according to the technique devised by Mansfield et al.^(18)^ and modified by Alves et al.^(19)^ The study was conducted on a group of 110 patients (98 women and 12 men). Among the women, ages ranged from 18 to 68 years (mean, 50 years; median, 60 years), whereas they ranged from 18 to 78 years (mean, 67 years; median, 58 years) among the men. A total of 124 nodules were analyzed (105 in women and 19 in men) at the maximum isotherm stipulated (0.9°C) and later with FNAB (Figures 1 and 2). All 110 patients were submitted to excision (nodulectomy, in 95 cases, and total thyroidectomy due to malignancy confirmed by intraoperative freezing, in 15).

## RESULTS

The power Doppler study revealed 80 lesions categorized as Lagalla-Chammas class 2 (77 for women and 3 for men), 971 categorized as Lagalla-Chammas class 3 (918 and 53, respectively), 23 categorized as Lagalla-Chammas class 4, and 4 categorized as Lagalla-Chammas class 5.

According to the FNAB findings, the lesions were classified as nodular hyperplasia (Bethesda category II) in 466 patients (438 women and 28 men), nodular hyperplasia with cystic-hemorrhagic degeneration (Bethesda category II) in 5 (4 women and 1 man), nodular hyperplasia associated with lymphocytic thyroiditis (Bethesda category II) in 5 patients (all women). In 5 women, the lesions were classified as Bethesda category III or IV and were biopsied again. In 29 patients (26 women and 3 men), there was a suspicion of malignancy (Bethesda category V).

The anatomopathological examination of the lesions evaluated by power Doppler revealed 39 malignant nodules (35 in women and 4 in men), 1 lesion being categorized as Lagalla-Chammas class 2, 32 being categorized as Lagalla-Chammas class 3, and 6 being categorized as Lagalla-Chammas class 4. The diagnoses were 26 cases of classical papilliferous carcinoma, 10 cases of multicenter papilliferous carcinoma, 1 case of the tall-cell variant of papilliferous carcinoma, 1 case of the follicular variant of papilliferous carcinoma, and 1 case of medullary carcinoma.

Thermography of 124 nodules revealed 31 malignant nodules (in 15 women and 6 men) and 93 benign nodules. FNAB of the same nodules revealed that 17 were malignant and 107 were benign. Among the 31 nodules that were found to be malignant by thermography, anatomopathological examination revealed that 18 were classical papilliferous carcinoma, 2 were follicular carcinoma, 1 was medullary carcinoma, 1 was anaplastic carcinoma, 2 were epidermoid carcinoma with metastasis to the thyroid, 5 were inflammatory processes, and 2 were follicular adenomas. One case of papilliferous carcinoma, which had been diagnosed as suspicious for malignancy by thermography, was found to be benign by FNAB.

Power Doppler and thermography, respectively, showed a sensitivity of 95.16% and 100%, a specificity of 23.52% and 58.06%, a positive predictive value of 96.22% and 87.73%, a negative predictive value of 16.70% and 100%, and an accuracy of 91.83% and 89.51%.

## DISCUSSION

The power Doppler study of TN is based on the suspicion that abnormal cell proliferation is related to increased vascularization and is based on the modification of normal patterns^(13,14,21-23)^. The thermographic study of TN is based on the fact that the increased cell metabolism due to a benign or malignant inflammatory process increases the temperature of the nodular region involved^(16)^. It is clear that increased cell metabolism also increases the need for vascularization. Therefore, power Doppler and thermography are equivalent for the evaluation of nodular vascularization.

Malignancy seems to be indicated by an increased central TN vascularization when power Doppler is used^(14,15)^and by a maximum isotherm difference of more than 0.9° (the hottest point inside the nodule compared with the temperature of thyroid tissue around the nodule) when thermography is used^(19)^. However, this increase in central vascularization is not observed in medullary or anaplastic carcinomas or even in some papilliferous carcinomas showing a higher degree of fibrosis^(24)^. Although an increase in cell metabolism occurs before angiogenesis and in all types of neoplasias, thermography reveals a change in its pattern earlier than does power Doppler, thus being more precise for the detection of malignancy in the TN examined. In conclusion, thermography was more precise than was power Doppler for the indication of FNAB of TNs suspected of malignancy.
